# An Intrahepatic Cholangiocarcinoma Patient with von Willebrand Disease Successfully Treated with Robotic Hepatectomy under von Willebrand Factor Supplementation

**DOI:** 10.70352/scrj.cr.25-0188

**Published:** 2025-07-31

**Authors:** Hiroto Chiba, Naoya Sato, Hiroshi Takahashi, Yoshiki Suzuki, Takayasu Azuma, Shigeyuki Tsukida, Makoto Muto, Yasuhide Kofunato, Teruhide Ishigame, Takashi Kimura, Akira Kenjo, Takayuki Ikezoe, Shigeru Marubashi

**Affiliations:** 1 Department of Hepato-Biliary-Pancreatic and Transplant Surgery, Fukushima Medical University, Fukushima, Fukushima, Japan; 2 Department of Hematology, Fukushima Medical University, Fukushima, Fukushima, Japan

**Keywords:** von Willebrand disease, hepatectomy, intrahepatic cholangiocarcinoma

## Abstract

**INTRODUCTION:**

Von Willebrand disease (VWD) is the second most common inherited coagulation disorder, and appropriate perioperative management is necessary when considering major surgery. There are few reports of patients with VWD who have undergone hepatectomy, especially minimally invasive hepatectomy. To our knowledge, this is the first reported case of a patient with VWD who successfully underwent robotic hepatectomy with von Willebrand factor (VWF) and factor VIII (FVIII) supplementation.

**CASE PRESENTATION:**

A 75-year-old female was referred to our hospital because of a liver tumor that was diagnosed during follow-up after hepatitis C treatment. She had also been diagnosed with VWD in her 30s. CT and MRI showed a 24-mm mass in segment 8 of the liver, bordered by the middle hepatic vein (MHV). To ensure safe perioperative management, replacement therapy with a VWF- or FVIII-containing concentrate was administered from preoperative day 1 to POD 14. Robotic extended segmentectomy (segment 8) was performed, with resection of the MHV. Liver parenchyma was dissected using the crush and clamp technique under the Pringle maneuver. Estimated intraoperative blood loss was 160 mL, and total operative time was 601 min. The patient needed 2 units of fresh frozen plasma on POD 1; however, no other transfusions, including red blood cells, were required. Although the patient presented with postoperative ascites and was treated with diuretics, she was discharged on POD 20 without any bleeding event. The final pathological finding was intrahepatic cholangiocarcinoma.

**CONCLUSIONS:**

We encountered a patient with intrahepatic cholangiocarcinoma and VWD who was successfully treated with anatomical hepatectomy by robotic-assisted laparoscopic surgery under perioperative replacement therapy with a VWF- or FVIII-containing concentrate. With appropriate perioperative management, major hepatectomy can be applied for VWD patients despite their high risk of postoperative hemorrhagic complications.

## Abbreviations

ALT
: alanine aminotransferase
APTT
: activated partial thromboplastin time
AST
: aspartate aminotransferase
FFP
: fresh frozen plasma
FVIII
: coagulation factor VIII
G-GTP
: gamma-glutamyl transferase
HCC
: hepatocellular carcinoma
MHV
: middle hepatic vein
RBC
: red blood cell
US
: ultrasonography
VWD
: von Willebrand disease
VWF
: von Willebrand factor

## INTRODUCTION

Von Willebrand disease (VWD) is an autosomal dominant or recessive hereditary disorder characterized by a bleeding tendency due to abnormalities in von Willebrand factor (VWF), which plays a crucial role in platelet adhesion and aggregation, as well as in the transport and stabilization of coagulation factor VIII (FVIII).^1)^ VWD is the second most common inherited coagulation disease, with a prevalence estimated at 60.3 per million.^2)^ Appropriate perioperative management is required to reduce the risk of bleeding, especially when major surgeries are planned.^3)^ Guidelines on the perioperative management of VWD recommend replacement therapy with VWF and FVIII during major surgery.^4,5)^

Minimally invasive surgery has been increasingly adopted in the field of hepatobiliary-pancreatic surgery, and the safety and efficacy of laparoscopic or robot-assisted liver resections have been reported.^6,7)^ However, even with minimally invasive surgery, strict perioperative management remains essential for patients with inherited bleeding disorders. Herein, we present a case of a liver tumor in a patient with a history of VWD. This is the first case report of robotic hepatectomy in a patient with VWD.

## CASE PRESENTATION

A 75-year-old female with VWD was referred to our institution due to a liver tumor that was found on routine abdominal ultrasonography (US) during follow-up care for hepatitis C. US-guided biopsy confirmed the diagnosis of intrahepatic cholangiocarcinoma. The detailed history of past treatments could not be confirmed; however, the patient had undergone antiviral therapy for hepatitis C and achieved seroconversion. Confact was administered at a dose of 1000 units daily for 3 consecutive days, on the day preceding, the day of, and the day following the liver biopsy. Coagulation parameters, including activated partial thromboplastin time (APTT), FVIII activity, and vWF activity, were monitored during this period.

She underwent laparotomy for an ectopic pregnancy at 31 years of age and required a perioperative transfusion. She was diagnosed with VWD and underwent routine annual checkups at a nearby clinic.

**Fig. 1:**
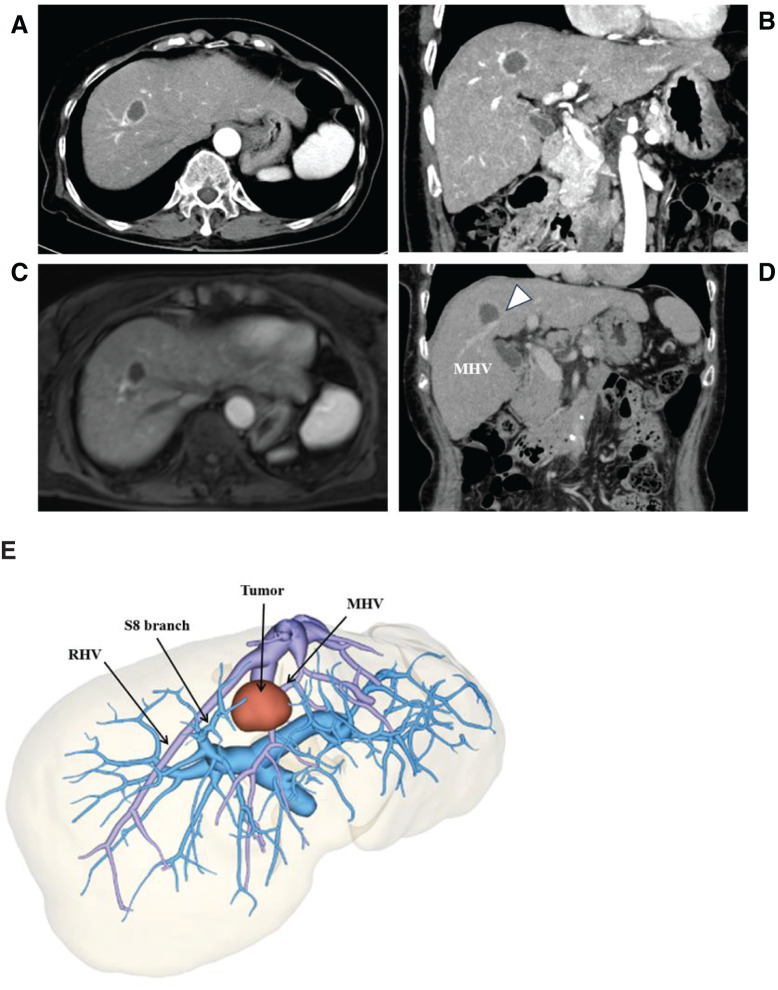
Findings of CT scan with contrast and 3D reconstruction. (**A**, **B**) Axial (**A**) and coronal (**B**) views of preoperative CT scan showing a 24-mm mass in segment 8 of the liver. (**C**, **D**) Axial (**A**) and coronal (**B**) views of the preoperative CT scan showing the tumor in segment 8 bordering the MHV (indicated by a triangle in the figure). (**E**) 3D reconstruction of the CT scan. MHV, middle hepatic vain; RHV, right hepatic vein

Contrast-enhanced US, CT, and MRI revealed a 24-mm mass in segment 8 of the liver, bordering the middle hepatic vein (MHV) (**Fig. 1**).

Preoperative laboratory data are shown in **Table 1**. In the coagulation test, APTT was slightly increased. The activity of VWF was 12%, and the VWF antigen was 43%. FVIII levels were also decreased. Biochemical analysis showed that aspartate aminotransferase (AST), alanine aminotransferase (ALT), total bilirubin, and gamma-glutamyl transferase were slightly increased. Complete blood count and tumor markers were unremarkable. The Child–Pugh score was grade A, and the patient had a history of hepatitis C treatment; however, the virus was not detected in a viral load test.

**Table 1:** Preoperative laboratory data

CBC		Biochemistry		Tumor markers	
WBC	3700/µL	Total protein	7.4 g/dL	CEA	3.0 ng/mL
RBC	4.28 × 10^6^/µL	Albumin	4.6 g/dL	CA19-9	14.5 U/mL
Hemoglobin	13.3 g/dL	BUN	12 mg/dL	AFP	2.4
Platelet	115 × 10^3^/µL	Creatinine	0.66 mg/dL	PIVKA-II	18
Coagulation		Total bilirubin	1.7 mg/dL		
PT	108.1%	Direct bilirubin	0.5 mg/dL	ICG R15	9%
APTT	39.3 s	AST	39 U/L	ICG K value	0.161
VWF activity	12%	ALT	34 U/L	Viral panel test	
VWF antigen	43%	LDH	213 U/L	HBs-Ag	Negative
Factor VIII	25.9%	ALP	26 U/L	HBs-Ab	Positive
		G-GTP	52 U/L	HBc-Ab	Positive
		ChE	259 U/L	HBV viral load	Negative
				HCV-Ab	Positive
				HCV PCR	Negative

AFP, alpha-fetoprotein; ALP, alkaline phosphatase; ALT, alanine transaminase; APTT, activated partial thromboplastin time; AST, aspartate transferase; BUN, blood urea nitrogen; CA19-9, carbohydrate antigen 19-9; CBC, complete blood count; CEA, carcinoembryonic antigen; ChE, cholinesterase; G-GTP, gamma-glutamyl transpeptidase; HBs-Ab, hepatitis B surface antibody; HBsAg, hepatitis B surface antigen; HBc-Ab, hepatitis B core total antibody; HBV, hepatitis B virus; HCV, hepatitis C virus; ICG R15, indocyanine green retention test at 15 min; LDH, lactate dehydrogenase; PCR, polymerase chain reaction; PIVKA-II, prothrombin induced by vitamin K absence or antagonist II; PT, prothrombin time; RBC, red blood cell; VWF, von Willebrand factor; WBC, white blood cell

**Fig. 2:**
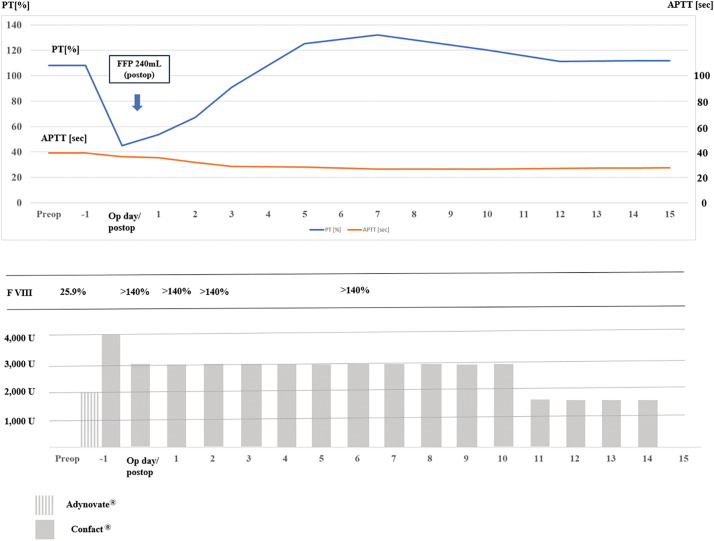
Administration schedule of VWF/FVIII concentrates. APTT, activated partial thromboplastin time; FVIII, factor VIII; PT, partial thromboplastin time; VWF, von Willebrand factor

Preoperative consultation with the hematology department was performed to ensure safe perioperative management. Regarding VWF and FVIII replacement, the details are as follows (**Fig. 2**): On the day before surgery, 2000 units of Adynovate (FVIII concentrate) and 4000 units of Confact (VWF/FVIII concentrate) were administered. On the morning of the surgery, FVIII activity was confirmed to be 140%, and an additional 1500 units of Confact were administered immediately before the start of the procedure. Postoperatively, 1500 units of Confact were administered, and this was followed by 3000 units daily from POD 1 to day 10, and 1500 units daily from days 10 to 14. Treatment was continued for 2 weeks after surgery. These replacement regimens were established in accordance with the guidelines for the invasive management of VWF-associated conditions.^4,5,8,9)^

In our department, minimally invasive hepatectomy is selected for the majority of cases, and robotic hepatectomy was chosen for this patient.

After induction of general anesthesia, the patient was placed in the semi-left lateral and reverse Trendelenburg positions. Robotic segmentectomy was performed using a 5-port setting. Before hepatic resection, cholecystectomy was performed, and a tourniquet was placed for the Pringle maneuver.

The right hepatic lobe was mobilized.

**Fig. 3:**
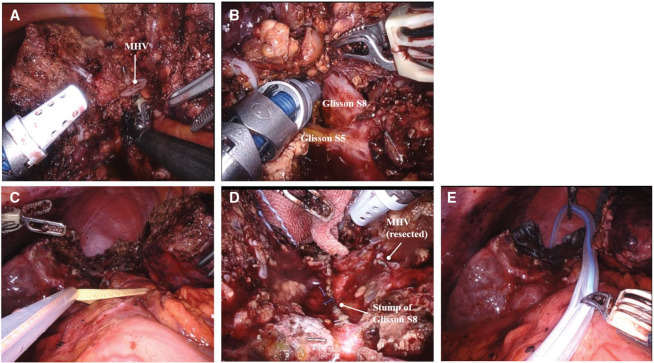
Intraoperative findings. (**A**) MHV, (**B**) encircling of the Glisson pedicle of S8, (**C**) view after completion of hepatic resection, (**D**) view after hepatic resection (resected vascular stump site), and (**E**) drain insertion. MHV, middle hepatic vein; S8, segment 8

We attempted the Glissonean approach for the Glissonean pedicle of segment 8. As the tumor partially extended into segment 4 and was in contact with the MHV, we first determined the transection line on the left side of the tumor within segment 4 under ultrasound guidance and initiated liver parenchymal transection in order to secure the Glissonean pedicle of segment 8. During this process, the peripheral portion of the MHV was resected. The Glissonean pedicle of segment 8 was exposed and ligated after transection at the boundary between segments 8 and 5. Following the ischemic demarcation between segment 8 and the posterior section of the liver, liver parenchymal transection was performed to complete the extended S8 segmentectomy. No tumor remnant was found. Parenchymal dissection was performed using the crush and clamp technique under the Pringle maneuver. Intraoperative findings are shown in **Fig. 3**. Estimated intraoperative blood loss was 160 mL, and total operative time was 601 min. The total duration of hepatic inflow occlusion with the Pringle maneuver was 235 min, while the robotic console time was 558 min.

**Fig. 4:**
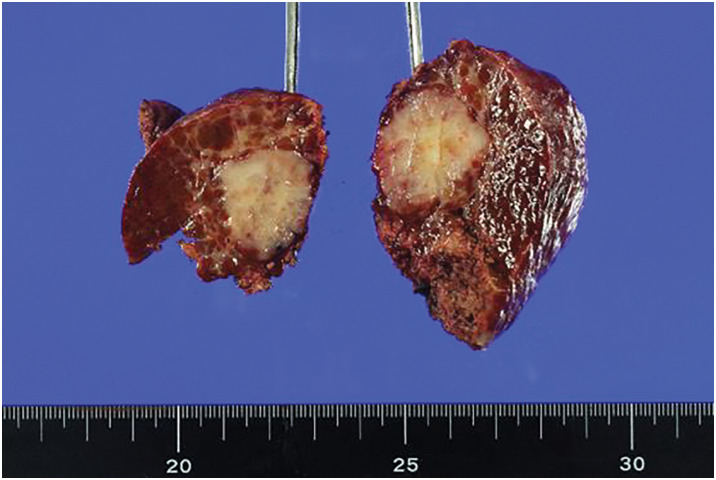
Specimen of the resected liver. A 25-mm, whitish tumor was found in the cross-section. The pathological diagnosis was intrahepatic cholangiocarcinoma.

Images of the specimen are shown in **Fig. 4**. A 25-mm tumor was observed in the cross-section of the specimen.

The final pathological finding was intrahepatic cholangiocarcinoma, with the following pathological details: poorly differentiated carcinoma with microscopic venous, portal venous, and biliary invasions. The METAVIR fibrosis score was F4 for the background-resected liver. The pathological stage was pT2N0M0 (UICC 8th edition^10)^).

Owing to a decrease in prothrombin time to 45% immediately after surgery, 2 units of fresh frozen plasma (FFP) were administered. However, no additional transfusion of FFP, red blood cells (RBCs), or platelets was required. The drain was removed on POD 5.

Postoperatively, weight gain due to ascites and subcutaneous edema was observed; thus, diuretics were started on POD 5. No other significant events occurred, and the patient was discharged on POD 20 to her home. There was no evidence of intrahepatic or extrahepatic metastasis at 6 months after surgery. During the postoperative course, the disease activity of VWD has remained stable without any signs of deterioration.

## DISCUSSION

VWD is a common inherited bleeding disorder and often causes hemorrhagic complications after invasive procedures. Perioperative replacement of VWF and FVIII is recommended to reduce the risk of bleeding, especially in major surgeries.^3,5,9)^

Following the guidelines for perioperative management of VWD by the Japanese Society of Thrombosis and Hemostasis, Confact F was started preoperatively in our case. In major surgeries, the guideline recommends the administration of VWF-containing concentrates, aiming for 100% target levels of VWF activity and FVIII activity after the primary dose. Subsequently, VWF and FVIII activities should be maintained above 50% for 7–10 PODs.^9)^

Hepatectomy in patients with inherited bleeding disorders, especially VWD, is rare. In Japan, a national survey on hepatobiliary and pancreatic surgery in patients with hemophilia was performed. In 26 hospitals with highly skilled specialist training facilities, 48 patients underwent hepatobiliary and pancreatic surgery between 2007 and 2017, and only 2 patients with VWD were included (without any surgical details in the article).^11)^ **Table 2** presents previous case reports on hepatectomy for VWD. The first English report of liver resection in a patient with VWD was by Kokudo et al. in 2014.^12)^ In the first domestic case report in Japan, Ishii et al. reported a case that underwent multiple hepatic partial resections in 2013.^1)^ In 2018, Kobayashi et al. reported 7 cases of hemophilia and 3 cases of VWD (one of which was the case previously reported by Kokudo et al.) at tertiary care hospitals in Japan and Switzerland.^13)^ They compared these 10 cases with 20 cases of hepatocellular carcinoma (HCC) (non-inherited bleeding disorder group) matched for analysis and found no significant differences in perioperative blood loss (730 vs. 820 mL, p = 0.748), major complications (10.0% vs. 5.0%, p = 0.605), or mortality rates (0% vs. 0%). Details of these 2 VWD cases are unknown. The first laparoscopic hepatectomy was reported by Sal et al. in 2024.^2)^ The case presented by Sal involved postoperative bleeding; however, it was treatable with interventional radiology. Two patients required perioperative RBC transfusion. All reported cases except that reported by Ishii et al. received VWF/FVIII concentrates. In the report by Ishii et al., only FVIII concentrate was administered. Compared with previous reports, intraoperative blood loss was lower in our case. This may be one of the advantages of the minimally invasive approach, as highlighted by Yoshimoto-Haramura et al.^11)^

**Table 2:** Previous studies of VWD cases undergoing hepatectomy for malignant liver tumors

Authors	Year	Age, sex	Procedure	Location	Number of tumors	Max diameter of tumor (mm)	VWF activity (%)	VWF antigen (%)	FVIII (%)	Intraoperative blood loss (mL)	RBC transfusion	Diagnosis	Postoperative complications	Replacement
Ishii et al.^1)^	2013	59F	Open, partial	S4, S6, S8	3	40	<6	52	—	1065	No	HCC	None	FVIII 22500 IU in total
Kokudo et al.^12)^	2014	50F	Open, right lobe	Rt. liver	1	160	—	—	6	2100	Yes	HCC	None	VWF/FVIII 2 h before Op: 60 U/kg 4 h after Op: 30 U/kg
Kobayashi et al.^13)^	2019	77F	Partial hepatectomy	S1	—	—	—	—	—	—	—	Malignant	Unknown	VWF/FVIII* −2 h before Op: 60 U/kg −4 h after Op: 30 U/kg −12 h after Op: 30 U/kg −30 U/kg × 2/day until POD7
		70F	Left hepatectomy	Lt. liver	—	—	—	—	—	—	—	Malignant	Unknown	VWF/FVIII* −2 h before Op: 60 U/kg −4 h after Op: 30 U/kg −12 h after Op: 30 U/kg −30 U/kg × 2/day until POD7
Sato et al.^3)^	2018	77M	Open, partial	S1, S5	2	15	6	24	83	484	No	HCC	No	VWF/FVIII 4800 IU in total
Sal et al.^2)^	2024	76M	Laparoscopic, partial	S7	1	37	30	97	71	2150	Yes	HCC	Bleeding (needed IVR)	VWF/FVIII 14000 IU in total
Current case		75F	Robotic sub-segmentectomy	S8	1	25	12	43	25.9	160	No	ICC	Ascites	VWF/FVIII

*12 h after Op: 30 U/kg, and 30 U/kg for every 12 h until 7 days after Op

F, female; FVIII, factor VIII; ICC, intrahepatic cholangiocarcinoma; IVR, interventional radiology; HCC, hepatocellular carcinoma; Lt., left; M, male; max, maximum; Op, operation; RBC, red blood cell; Rt., right; S, segment; VWD, von Willebrand disease; VWF, von Willebrand factor.

Few cohort studies have investigated the safety of laparoscopic procedures in patients with inherited bleeding disorders. However, a study by Denzer et al. in 2003 reported on mini-laparoscopic liver biopsy in 61 patients with marked coagulopathy, including 5% with VWD.^14)^ In this study, no patients experienced bleeding complications. Two case reports of laparoscopic hepatectomy in patients with hemophilia were found in PubMed. Matsuda et al. reported performing laparoscopic left lateral segmentectomy in a 60-year-old male with hemophilia A while administering replacement therapy; however, postoperative intra-abdominal hemorrhage occurred.^15)^ In 2023, Tsukagoshi et al. reported performing laparoscopic hepatectomy (both partial liver resections) in 2 HCC patients with hemophilia, with no postoperative complications observed in either case.^16)^ Additionally, Goka et al. reported a successful case of radiofrequency ablation for HCC in a patient with hemophilia A.^17)^

Generally, minimally invasive surgery is associated with less blood loss than open surgery.^10)^ Several studies have shown that robotic liver resection can achieve comparable or, in some cases, even superior outcomes in terms of intraoperative blood loss compared to laparoscopic liver resection.^18–21)^ The advantage of robotic surgery lies in its ability to retain the benefits of minimally invasive techniques while offering the freedom of movement comparable to that of open surgery.^22)^ Especially in anatomical resections such as segmentectomy, encircling and dissecting the Glissonean pedicle are essential for the success of surgery. Robot-assisted surgery enables these complex maneuvers to be performed effectively, thanks to the multi-joint functionality of the robotic arms.

However, a significant concern is the difficulty in maintaining a clear operative field once bleeding occurs during the procedure. This is especially important in cases with a bleeding tendency, in which careful consideration is required when applying minimally invasive techniques, particularly during liver resection. There have been limited reports on laparoscopic or robot-assisted liver surgery in patients with inherited bleeding disorders, including VWD. Although the safety of such approaches should be further investigated using large-scale studies, it is noteworthy that, with the assistance of hematologists and anesthesiologists, we performed robot-assisted liver resection without bleeding complications by managing the patient perioperatively using VWF- and FVIII-containing products.

## CONCLUSIONS

We encountered a patient with intrahepatic cholangiocarcinoma (ICC) and VWD who successfully underwent an anatomical hepatectomy via robotic-assisted laparoscopic surgery under perioperative replacement therapy with a VWF- or FVIII-containing concentrate. With appropriate perioperative management, anatomical hepatectomy can be performed in patients with VWD despite the high risk of postoperative hemorrhagic complications. Such high-risk cases should be managed at high-volume centers where specialists in anesthesiology and hematology are available.

## ACKNOWLEDGMENTS

We would like to thank Editage (www.editage.jp) for the English language editing.

## DECLARATIONS

### Funding

Not applicable.

### Authors’ contributions

Manuscript preparation: Hiroto Chiba

Supervision of manuscript creation: Naoya Sato, Takashi Kimura, Akira Kenjo, and Shigeru Marubashi

Revision of the manuscript: Yoshiki Suzuki, Hiroshi Takahashi, Takayasu Azuma, Shigeyuki Tsukida, Makoto Muto, Naoya Sato, Yasuhide Kofunato, Teruhide Ishigame, Takashi Kimura, Akira Kenjo, Takayuki Ikezoe, and Shigeru Marubashi

All authors have read and approved the manuscript.

### Availability of data and materials

Not applicable.

### Ethics approval and consent to participate

This work does not require ethical considerations or approval.

Informed consent to participate in this study was obtained from the patient.

### Consent for publication

Consent for the publication of the case details was obtained from the patient.

### Competing interests

The authors declare no conflicts of interest.
